# Hysteroscopic Morcellation of Submucous Myomas: A Systematic Review

**DOI:** 10.1155/2017/6848250

**Published:** 2017-08-29

**Authors:** Salvatore Giovanni Vitale, Fabrizio Sapia, Agnese Maria Chiara Rapisarda, Gaetano Valenti, Fabrizia Santangelo, Diego Rossetti, Benito Chiofalo, Giuseppe Sarpietro, Valentina Lucia La Rosa, Onofrio Triolo, Marco Noventa, Salvatore Gizzo, Antonio Simone Laganà

**Affiliations:** ^1^Unit of Gynecology and Obstetrics, Department of Human Pathology in Adulthood and Childhood “G. Barresi”, University of Messina, Messina, Italy; ^2^Department of General Surgery and Medical Surgical Specialties, University of Catania, Catania, Italy; ^3^Department of Obstetrics and Gynecology, University of Naples “Federico II”, Naples, Italy; ^4^Department of Maternal and Child Health, Gavardo Hospital, Brescia, Italy; ^5^Unit of Psychodiagnostics and Clinical Psychology, University of Catania, Catania, Italy; ^6^Department of Woman and Child Health, University of Padua, Padua, Italy

## Abstract

Hysteroscopic surgery is the actual gold standard treatment for several types of intrauterine pathologies, including submucous myomas (SMs). To date, the availability of Hysteroscopic Tissue Removal systems (HTRs) opened a new scenario. Based on these elements, the aim of this article is to review the available evidence about HTRs for the management of SMs. We included 8 papers (3 prospective studies and 5 retrospective studies). A total of 283 women underwent intrauterine morcellation of SM: 208 were treated using MyoSure and 75 using Truclear 8.0. Only 3 articles reported data about procedures performed in outpatient/office setting. Only half of the included studies included type 2 SMs. HTRs significantly reduced operative time compared to traditional resectoscopy in some studies, whereas others did not find significant differences. Despite the availability of few randomized controlled trials and the cost of the instrument, according to our systematic review, the use of HTRs seems to be a feasible surgical option in terms of operative time and complications. Nevertheless, the type of SM still remains the biggest challenge: type 0 and 1 SMs are easier to manage with respect to type 2, reflecting what already is known for the “classic” hysteroscopic myomectomy.

## 1. Introduction

The progressive improvement of hysteroscopic instruments and the standardization of techniques allowed feasible and daily management of submucous myomas (SMs). Hysteroscopic myomectomy is usually performed with a progressive slicing of the intracavitary portion of the SM, a subsequent “cold loop” pushing of the intramural part (to preserve the pseudocapsule), and, finally, a slicing resection of it [1–3]. As was widely reported, a careful and conscious management of uterine myomas improves not only symptoms, but also fertility outcomes [4, 5].

To date, the availability of Hysteroscopic Tissue Removal systems (HTRs) opened a new scenario for hysteroscopic myomectomy: indeed, the learning curve for resectoscopic management of SM is challenging for both the residents and specialists and may lead also to severe complications [6]. In this regard, HTRs may reduce the learning curve and complication rate of hysteroscopic myomectomy for SM with respect to traditional resectoscopy.

The use of morcellators in gynecologic surgery started for myomectomy and hysterectomy first in laparoscopy; however in 2014 the U.S. Food and Drug Administration warned against the use of laparoscopic power morcellators for the risk of spreading an unsuspected cancer [7]. Nevertheless, this Safety Communication does not affect HTRs. HTRs consist of 2 metal, hollow, rigid, and disposable tubes with a wide range of diameters adaptable to the use of 5 to 9 mm hysteroscopes. Different HTRs are commercially available: Truclear 8.0 (Medtronic, Minneapolis, Minnesota), Truclear 5C (Medtronic, Minneapolis, Minnesota), and MyoSure (Hologic, Marlborough, Massachusetts). As recently summarized by Noventa et al. [8], Truclear 8.0 has a diameter of 8 mm and is introduced into the uterine cavity with a 9 mm rigid sheath; Truclear 5C hysteroscopy system incorporates a 2.9 mm rotatory-style blade through a 5 mm, 0° hysteroscope; MyoSure is introduced into the uterus through a 6 or a 7 mm, 0°, continuous flow hysteroscope. All these devices work with physiologic saline solution as distension and irrigation media, instead of the electrolyte-free solutions used for monopolar high-frequency resectoscopy.

Considering that data published so far do not allow drawing a firm conclusion, the aim of this article is to review the available evidence about the role of HRTs for the management of SMs.

## 2. Materials and Methods

We performed the database search on Scopus, PubMed/MEDLINE, and Science Direct. We searched with “Hysteroscopic Tissue Removal system”, “Intrauterine morcellator”, and “Hysteroscopic morcellator”. We considered eligible original articles (randomized, observational, retrospective studies) about SM management through the use of HTRs, excluding case reports and video articles, published between 2000 and 2016 in English and French languages.

Titles and/or abstracts of retrieved articles were screened independently by two authors (F. S. and G. V.) to identify studies that potentially meet the inclusion criteria outlined above. The full texts of these potentially eligible studies were retrieved and independently assessed for eligibility by other two authors (B. C. and A. M. C. R.). Any disagreement between them over the eligibility of particular studies was resolved through discussion with a third author (A. S. L.). A standardized, prepiloted form was used to extract data from the included studies for assessment of study quality and evidence synthesis. We selected information about study design, type of SMs, type of HTRs, operative time, fluid balance, and operative outcomes. Studies providing ambiguous or insufficient data or not quantifiable outcomes were excluded from the current analysis.

## 3. Results

**Figure 1:**
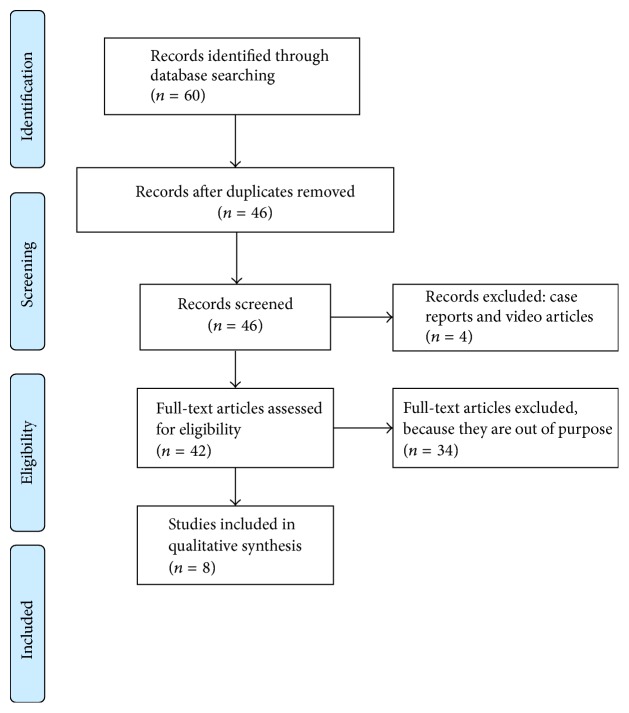
Searching strategy. Adapted from Moher D., Liberati A., Tetzlaff J., Altman D. G., and The PRISMA Group. Preferred Reporting Items for Systematic Reviews and Meta-Analyses: The PRISMA Statement.

Using the reported search strategy, we identified 19 items for “Hysteroscopic Tissue Removal system”, 14 items for “Intrauterine morcellator”, and 27 items for “Hysteroscopic morcellator”. After exclusion of 14 duplicates, we screened 46 items and further excluded 4 of them because they were case reports and/or video articles. The remaining 44 items were selected and each full text was carefully evaluated, in order to select only relevant information (hysteroscopic morcellation of submucous myoma). Since 34 full texts were out of purpose, in the current systematic review, we included the remaining 8 papers [9–16] that met the abovementioned inclusion criteria (the search strategy is summarized in Figure 1). As summarized in Table 1, 3 were prospective studies [9–11] and 5 were retrospective [12–16]. In all articles, patients' mean age was above 40 years. The authors used MyoSure in 5 articles [9, 11–14] and Truclear 8.0 in the remaining 3 [10, 15, 16]. The total retrieved cases of SM treated with HTRs were 283: 208 cases were performed using MyoSure devices, whereas 75 were performed using Truclear 8.0. Interestingly, only two articles reported data about procedures performed in outpatient/office setting [9, 13], whereas all the other collected cases were performed in operating theatre. Among the included studies, less than half [11, 13, 14] included data about type 2 SM. All the data about operative time, fluid deficit, complications, and main outcomes are reported in Table 1 and will be discussed in the next section.

## 4. Discussion

Operative time data were available in half of the included studies [10, 12, 14–16] and, in any case, were extremely variable: minimum average time was 10.6 min, for van Dongen et al. [10]; maximum was 36.6 min for Lee and Matsuzono [12]. The first data seems more reliable because it came from a randomized controlled trial, while other available data about operative times came from retrospective studies. Similarly, fluid deficit data were available only in 6/8 articles: they are almost overlapping (or at least comparable) in only 4 studies [10, 13, 15, 16], between an average of 409 mL in the only RCT [10] and 660 mL in a retrospective study [15]; only in 2 articles [11, 12], fluid deficit was higher, between an average of 880 mL [11] and 1800 mL. The article from Arnold et al. [11] that reports a fluid deficit of 880 mL is a prospective study with a cohort of 102 cases. In the article of Rubino and Lukes [9], fluid deficit for all patients is not available, but a deficit nearly 4 L has been reported at least in one patient, which is contrary to hysteroscopic fluid management guidelines [17]. In this regard, usually a maximum fluid deficit of 1000 mL is recommended, but with the advent of bipolar electrosurgical systems in traditional resectoscopy and in HTRs a deficit of 2500 mL of isotonic solution is well tolerated by healthy women [18]. The mean operative time is 22,6 min and the mean fluid deficit is 730 mL. All the articles did not report significant intra- or postoperative complications. These data are of paramount importance, since it was clearly demonstrated that bleeding rate is associated with the degree of SM intracavitary development [19]. Only 3 retrospective studies and one small cohort prospective study compared resectoscopy to HTRs, so data are extremely limited. In some studies that compared intrauterine morcellation to resectoscopy, HTRs significantly reduced operative time [10, 12, 15], whereas others did not find significant differences [14]. In addition, the overall complete resection rate of SMs using HTRs seems to be comparable to resectoscopy [11, 14]. In detail, authors of the retrospective studies reported reduced operative times with HTRs compared to resectoscopy; Lee and Matsuzono found no significant differences in overall patient satisfaction and improvement in hemoglobin level between the two methods at 3-month follow-up [12], whereas Hamidouche et al. do not signaled differences for mean operative time, resection rate, adverse events, and intrauterine postoperative adhesions in a larger cohort (34 myomas) with respect to the precedent study (13 myomas) [14]. Finally, recent data suggest that both Uterine Fibroid Symptom-Quality of Life and Health-Related Quality of Life improve significantly 12 months after myomectomy using HTRs [9].

## 5. Conclusion 

Despite the introduction of HTRs in the clinical practice several years ago, published data about their use for the management of SMs are so far extremely limited, especially because this technique was not so attractive for surgeons, probably due to the large consent gained through the time by traditional resectoscopy. The available studies differ significantly regarding methodology and inclusion and exclusion criteria, and these elements clearly affect the comparison of intra- and postoperative outcomes among them. Despite these clear limitations, our overview allows us to confirm a good feasibility of HTRs use for type 0 and type 1 SMs and, similarly to what happens for “classic” resectoscopic myomectomy, a more difficult procedure for type 2 SMs (although there are reports of type 2 SMs managed in outpatient/office setting). The above reported data suggest that HTR is safe and does not increase the complication rate and postoperative adhesions with respect to resectoscopy, especially for in-training hysteroscopists due to a shorter learning curve. Several studies reported a significant reduction of operative time using HTRs, which may allow a consequent reduction of fluid deficit and avoid its overload. Finally, the medium-term follow-up seems to show good results after HTRs use, especially in terms of patient's satisfaction. The clear disadvantage is the higher cost, considering that the complete treatment of type 2 SMs often requires both HRT and resectoscope in the operating theatre, since HRTs are able to remove the SM once it is completely translated into the uterine cavity.

Nevertheless, HRTs are not so diffused worldwide, therefore data are not enough robust to draw firm conclusion about intrauterine morcellation of SMs, even due to important differences in the design of available studies. In particular, future randomized controlled trials with large cohorts and long-term follow-up with an adequate statistical power should investigate the efficacy of HRTs with respect to “classic” resectoscopic myomectomy, taking into account a possible subanalysis according to number, size, and type of SMs. In addition, we solicit accurate Health Technology Assessment in order to clarify the cost-effectiveness and impact of healthcare policy of HRTs, especially in office setting.

## Figures and Tables

**Table 1:** Relevant data of retrieved studies.

Authors, year	Type of study	Enrolled cases	Mean age	Type of myomas	Type of HTRs	Mean operative time (min)	Mean fluid deficit (mL)	Main findings
Rubino and Lukes, 2015 [9]	Randomized, prospective, comparative setting clinical trial	42 myomas	41.4 (overall, including also other pathologies)	Type 0 or 1 myomas > 1.5 and <3.0 cm	MyoSure	NA	NA	(1) 28 myomectomies were performed in office setting and 14 in ambulatory surgical center setting. (2) The mean percentage of pathology removed was 95.9 ± 6.8%. (3) Both Uterine Fibroid Symptom-Quality of Life and Health-Related Quality of Life improved significantly 12 months after procedure. (4) A fluid deficit of nearly 4 Lt was reported in at least one patient.

van Dongen et al., 2008 [10]	Prospective randomized controlled study	10 myomas	49	Type 0, type 1	Truclear 8.0	10.6	409	The use of the HTRs reduced operating time more than 8 min in comparison to conventional resectoscopy.

Arnold et al., 2016 [11]	Prospective cohort study	102 myomas	43	29 type 0, 38 type 1, 17 type 2, 18 not documented	MyoSure	NA	880	63 complete resection of pathology at the end of the procedure: 18 type 0, 27 type 1, and 11 type 2 myomas.

Lee and Matsuzono, 2016 [12]	Retrospective study	13 myomas	NR	2 patients with myoma protrusion < 60%, 11 patients > 60%; 9 small (≤3 cm) myomas, 4 large (>3 cm) myomas	MyoSure	36.6	1005	(1) No significant differences in overall patient satisfaction and improvement in hemoglobin level between intrauterine morcellation and resectoscopy at 3-month follow-up. (2) HTRs significantly reduced operative time.

Rajesh and Guyer, 2015 [13]	Retrospective study	17 myomas	58.6	Type 0, type 1, and type 2; size 1–5 cm	MyoSure	NA	495.3	(1) All patients had successful removal of pathology apart from two partial myomectomies (calcified fibroids) and one failed MyoSure for patulous cervix. (2) No complications occurred. (3) Intrauterine morcellation is feasible also in outpatient setting.

Hamidouche et al., 2015 [14]	Retrospective study	34 myomas	40.8	Type 0, type 1, and type 2	MyoSure	30.8	NA	No significant differences for mean operative time, complete resection rate, adverse events, and postoperative adhesion between HTRs and bipolar loop resection.

Emanuel and Wamsteker, 2005 [15]	Retrospective study	28 myomas	44.6	15 type 0, 13 type 1	Truclear 8.0	16.4	660	(1) Significant reduction of operative time for HTRs compared to resectoscopy. (2) No complications.

Hamerlynck et al., 2011 [16]	Retrospective study	37 myomas	45	Type 0, type 1	Truclear 8.0	18.2	440	(1) All procedures were uneventful. (2) Implementation of the HTRs for removal of type 0 and 1 myomas ≤ 3 cm, and removal of polyps appears safe and effective.

HTRs: Hysteroscopic Tissue Removal systems; NA: not available.
